# AdHu5Ag85A Respiratory Mucosal Boost Immunization Enhances Protection against Pulmonary Tuberculosis in BCG-Primed Non-Human Primates

**DOI:** 10.1371/journal.pone.0135009

**Published:** 2015-08-07

**Authors:** Mangalakumari Jeyanathan, Zhongqi Shao, Xuefeng Yu, Robin Harkness, Rong Jiang, Junqiang Li, Zhou Xing, Tao Zhu

**Affiliations:** 1 McMaster Immunology Research Centre and Department of Pathology & Molecular Medicine, McMaster University, Hamilton, Ontario, Canada; 2 Tianjin CanSino Biotechnology, Inc., Tianjin, China; National Jewish Health, UNITED STATES

## Abstract

Persisting high global tuberculosis (TB) morbidity and mortality and poor efficacy of BCG vaccine emphasizes an urgent need for developing effective novel boost vaccination strategies following parenteral BCG priming in humans. Most of the current lead TB vaccine candidates in the global pipeline were developed for parenteral route of immunization. Compelling evidence indicates respiratory mucosal delivery of vaccine to be the most effective way to induce robust local mucosal protective immunity against pulmonary TB. However, despite ample supporting evidence from various animal models, there has been a lack of evidence supporting the safety and protective efficacy of respiratory mucosal TB vaccination in non-human primates (NHP) and humans. By using a rhesus macaque TB model we have evaluated the safety and protective efficacy of a recombinant human serotype 5 adenovirus-based TB vaccine (AdHu5Ag85A) delivered via the respiratory mucosal route. We show that mucosal AdHu5Ag85A boost immunization was safe and well tolerated in parenteral BCG-primed rhesus macaques. A single AdHu5Ag85A mucosal boost immunization in BCG-primed rhesus macaques enhanced the antigen–specific T cell responses. Boost immunization significantly improved the survival and bacterial control following *M*.*tb* challenge. Furthermore, TB-related lung pathology and clinical outcomes were lessened in BCG-primed, mucosally boosted animals compared to control animals. Thus, for the first time we show that a single respiratory mucosal boost immunization with a novel TB vaccine enhances protection against pulmonary TB in parenteral BCG-primed NHP. Our study provides the evidence for the protective potential of AdHu5Ag85A as a respiratory mucosal boost TB vaccine for human application.

## Introduction

Pulmonary tuberculosis (TB) remains a leading infectious cause of global morbidity and mortality, causing approximately 1.5 million deaths and 9 million new cases each year [1]. An estimated 1/3 of the world population is latently infected by *Mycobaterium tuberculosis* (*M*.*tb)* and 5–10% of these people will develop active TB some time in their lives. BCG, the only TB vaccine currently in use, is given once parenterally via the skin shortly after birth in most countries. While BCG effectively protects against disseminated childhood TB, it has failed to control adolescent and adult pulmonary TB [2,3]. Thus, there is an urgent need to develop novel TB vaccines that can be used for effective boost vaccination following parenteral BCG priming in humans [3,4].

It is increasingly realized that in addition to the formulation of candidate vaccines, the new immunization strategy needs to take the route of vaccination into consideration [5,6]. This realization is based on recent findings that *M*.*tb* has evolved mechanisms to significantly slow down the appearance of T cell immunity in the lung of even the parenteral-BCG vaccinated hosts [7,8,9]. Such a delay or “immunological gap” gives *M*.*tb* a foothold in the lung before protective immunity is established, which likely represents an important mechanism underlying the ineffectiveness of parenteral TB vaccination [8,9]. Indeed, it has recently been reported that an intradermally delivered MVA-based TB vaccine fails to enhance protective immunity in BCG-primed infants in a major milestone phase 2b trial [10]. Recent evidence from others and us has identified the respiratory mucosal route of vaccination to be the most effective way to address the immunological gap in T cell immunity in the lung, and to effectively enhance protection against pulmonary TB [3,5,6]. However, almost all novel TB vaccine formulations in the TB vaccine pipeline have been or are being evaluated via a parenteral route for human application [2,3,4]. In this regard, protein- and replicating mycobacterial organism-based TB vaccines may not be amenable to respiratory mucosal applications due to a limited choice of immune adjuvants and safety concerns. By comparison, recombinant, replication-deficient virus-vectored TB vaccine platforms such as MVA- and adenovirus (Ad)-based candidates hold great promise for respiratory mucosal vaccination [3]. Indeed, recombinant MVA-, Ad35- and Ad5-based TB vaccines have all been evaluated in humans following a parenteral route of vaccination with demonstrated safety and immunogenicity [10,11,12]. However, none of these candidates had been evaluated in human clinical trials via the respiratory mucosal route until recently [13].

Investigation of candidate TB vaccines in non-human primate (NHP) models of pulmonary TB represents a critical step towards clinical studies to assess their protective potential for human applications [14]. To date, while a number of novel TB vaccine candidates have been evaluated in NHP models following parenteral vaccination [15,16,17,18], similar to the status of human studies, only MVA- and Ad35-based TB vaccines have recently been evaluated via the respiratory mucosal route in NHP models [19,20]. However, there is a lack of evidence to indicate that any of these aerosol-delivered vaccines improved immune protection against pulmonary *M*.*tb* challenge [19,20].

As one of the most promising virus-based TB vaccines developed by us, AdHu5Ag85A, the human adenovirus serotype 5 expressing the mycobacterial secreted antigen Ag85A, has been shown to confer robust protection against TB, particularly after respiratory mucosal delivery, in a number of preclinical models including the mouse, guinea pig, goat and cattle [3,5,21]. Recently, we have also reported that the intramuscular delivery of AdHu5Ag85A vaccine to healthy human volunteers is safe and immunogenic despite the pre-existing anti-AdHu5 backbone immunity [12]. In the current study, we have investigated the safety, immunogenicity and protection of this vaccine following respiratory mucosal delivery in a NHP model. We have found that respiratory mucosal boosting with AdHu5Ag85A via intratracheal inoculation or aerosol is safe and immunogenic in parenteral BCG-primed rhesus macaques. Of importance, the respiratory mucosal boost vaccination enhanced protection and reduced lung tissue immunopathology and associated clinical outcomes. Thus, we provide the evidence that a respiratory mucosal boost vaccination using a recombinant virus-based TB vaccine enhances protection in the lung of a NHP model of pulmonary TB. Our study provides important proof of principle to support further clinical development of AdHu5Ag85A and other viral-vectored vaccines for respiratory mucosal vaccination in humans. It also holds implications in developing mucosal vaccination strategies against other respiratory pathogens.

## Materials and Methods

### Ethics statement

The work described in this study was carried out in Wuhan University via a service contract. All animals were housed in the Animal Bio-Safety Level III (ABSL-III) Laboratory located at the Wuhan University School of Medicine. The housing and animal care procedures were in compliance with the Chinese guidelines for animal experiments (Laboratory animal Requirements of environment and housing facilities GB 14925–2010, China; Regulations on administration of laboratory animals, Ministry of Science and Technology, 1988, China) and with the 8th Guide for the Care and Use of Laboratory Animals of Association (National Research Council, 2011). The ABSL-III Laboratory is certified by the Association for Assessment and Accreditation of Laboratory Animal Care International (AAALAC International). Institutional Animal Care and Use Committee of Wuhan University School of Medicine approved all study protocols and procedures prior to starting this project (project license number S01312122E). The animal welfare and enrichment were provided according to the recommendations of the Weatherall report. The animals were individually housed in stainless steel wire-bottomed cages (80cm x 80cm x 80cm dimension) with sufficient space supplied with a commercial monkey diet and water and twice daily with fresh fruit in an air-conditioned room and monitored by a computer-based system. After infection, animals were also monitored twice daily by experienced staff. Furthermore, additional enrichment including rings, perches, forage boxes and puzzle-feeders was also provided. Animal health was monitored daily by the animal care and veterinary personnel. Pre-defined humane endpoints including depressed or withdrawn behavior, abnormal respiration rates, serious loss of appetite, severe body weight loss and severe abnormal chest x-ray changes were applied to reduce discomfort in this study. Serious loss of appetite was defined as no food intake during at least two meals. Severe body weight loss was defined as a 20% weight loss in three consecutive weeks compared to the body weight before *M*.*tb* infection. Animals were euthanized first by anesthesia with ketamine (i.m 10 mg/kg) and then by a lethal dose injection of sodium pentobarbital (i.v 100 mg/kg).

### Experimental animals and handling

Twenty-eight Chinese rhesus macaques, all males, 2–3 years old, 3–5 kg weight, used in the study were purchased from Hubei Tianqin Non-Human Primates Breeding and Research Center, Hubei, China. Prior to experimentation, all animals were screened strictly according to the National Standard (GB14922.2–2001) to ensure that they were free of specific pathogens, including mycobacteria [22]. Animals were given experimental serial numbers (WCS01 to WCS28). Animals were housed and fed as described previously [22]. Animals were anesthetized with ketamine (10 mg/kg) before handling. Blood for immune monitoring was collected into Vacutainer CPT tubes (BD Biosciences) and blood for standard hematology was collected into EDTA coated tubes by venipuncture. Serum was prepared for standard clinical biochemistry.

### Respiratory mucosal or parenteral immunization with AdHu5Ag85A or BCG vaccine

*M*.*bovis* BCG pasture vaccine (SSI, Copenhagen, Denmark; Batch No. 111005A) at the concentration of 7.8x10^6^ CFU/mL and Sautons diluent (SSI, Copenhagen, Denmark; Batch No. 313658713) were provided by Aeras Global TB Vaccine Foundation. Anaesthetized animals were immunized with BCG in the back via intradermal route with 100μl containing 5x10^5^CFU. AdHu5Ag85A vaccine (Batch No. FP-01, Lot No. 03) was prepared in the GMP facility at Robert E. Fitzhenry Vector Laboratory, McMaster University, Canada. AdHu5Ag85A vaccine was diluted in saline to appropriate concentration. Anaesthetized animals were immunized with 1.0x10^9^ PFU AdHu5Ag85A in 1ml via intramuscular (im) route in the leg or 1.0x10^9^ PFU AdHu5Ag85A in 2 ml via intratracheal instillation (it) or 1.0x10^9^ PFU AdHu5Ag85A in 0.5 ml buffer for aerosol delivery (aerosol). Aerosol delivery device, including an aeroneb solo nebulizer and a control module, was kindly provided by Aerogen. The masks were obtained from King Systems and modified for use in monkeys. For aerosol delivery animal was placed in dorsal recumbence on the procedure table after anesthetisation, AdHu5Ag85A vaccine was added into the chamber of the aerosol device and the modified mask connected to the device was placed on the face of the monkey.

### *M*.*tuberculosis* preparation and lung infection via bronchoscopy

A stock of the *M*.*tb* Erdman strain obtained from the Food and Drug Administration (Bethesda, MD, United States) through Aeras Global TB Vaccine Foundation was used for infection. A bacterial suspension of *M*.*tb* at the concentration of 12.5 CFU/mL was prepared by serial dilution with saline. Animals were anesthetized with ketamine (10 mg/kg) followed by an injection of atropine (0.04 mg/kg) to alleviate excessive salivation and maintain heart rates. 1 mL of lidocaine (0.1%) was sprayed into the mouth prior to the bacterial injection. 2 mL of the diluted bacterial suspension containing 25 CFU was delivered to the right lung through a flexible fiber-optic bronchoscope (2.8-mm outer diameter, OLYMPUS, Japan) as previously described [22,23]. Three minutes after bacterial inoculation, animals were returned to their cages in a right lateral recumbent position and were closely monitored for breathing pattern and heart rates until they were fully recovered from the anesthesia. The challenge dose was verified by plating the bacterial suspensions from the last three dilutions on the 7H10 plates for CFU determination. Animals were euthanized at the pre-determined endpoint of the study. Animals that reached the humane endpoint prior to pre-determined endpoint were humanely euthanized.

### Mycobacaterial colony forming assay

Tissue specimens from each individual lung lobe and spleen were selected randomly as described previously [24]. The tissue specimens from each lobe were pooled and weighted (about 1g per lobe) and then minced and homogenized in PBS using a tissue homogenizer. A 100 μl of each 10-fold serial diluted homogenized tissues were plated on 7H10-OADC agar plates and incubated at 37°C. The numbers of CFU on the agar plates were counted 3–5 weeks later.

### Fresh PBMC IFN-γ ELISPOT assay

ELISPOT was performed on freshly isolated PBMCs with MONKEY IFN-γ ELISPOT KIT (U-CyTech, Utrecht) according to the manufacturer’s instructions. PBMCs were isolated and stimulated as previously described [12]. PBMCs were washed and resuspended in RPMI 1640 supplemented with 10% fetal bovine serum (FBS) and 1% L-glutamine. PBMCs (0.3 x10^6^ per well) were plated in the presence of antigen in duplicate and incubated for 22 hours. Antigens used for stimulation included PPD (10 μg/ml), rAg85A (5 μg/ml), and each of six pools of 7 to 10 overlapping Ag85A peptides (10 μg/ml of each peptide). A complete peptide library used for stimulation consisted of the 15-mer peptides with a 10-mer overlap spanning the Ag85A protein expressed by AdHu5Ag85A. Upon incubation, wells were washed and processed for ELISPOT detection. IFN-γ+ cells were enumerated with CTL-Immunospot microanalyzer (Cellular Technology Ltd.). The average number of IFN-γ+ cells, if any, in the unstimulated wells was subtracted from that of stimulated wells, and the resulted spot counts were used for analyses. Stimulation with the peptide pools might have potentially resulted in a T cell reactive to the 10-mer overlapping regions across the two adjacent pools, being counted twice.

### Necropsy and pathological evaluation

All the animals humanely culled were anaesthetized with ketamine (10 mg/kg, i.m.) and terminated by injecting a lethal dose of sodium pentobarbital (100 mg/kg) intravenously and necrotized immediately in an ABSL-III facility. Gross pathology was evaluated and recorded using a predefined scoring system [25] based on the number and extent of lesions present in the major organs, including each of lung lobes, spleen, liver, kidney, and lymph nodes.

For histopathological examination, tissue samples of lung selected randomly using stereology-guided approach [24] were preserved in 10% formalin. Routinely processed and paraffin embedded tissues were sectioned at 4 μm thickness and stained with hematoxylin and eosin (H&E). The sections were then examined by a certified pathologist blinded to the treatment groups for histopathological changes under a light microscope (Olympus DP73, Japan). Three slides from each tissue sample of different lobes were examined and scored according to granulomatous lesions' number/size and cellular/tissue composition.

### Chest X-rays

**Fig 1:**
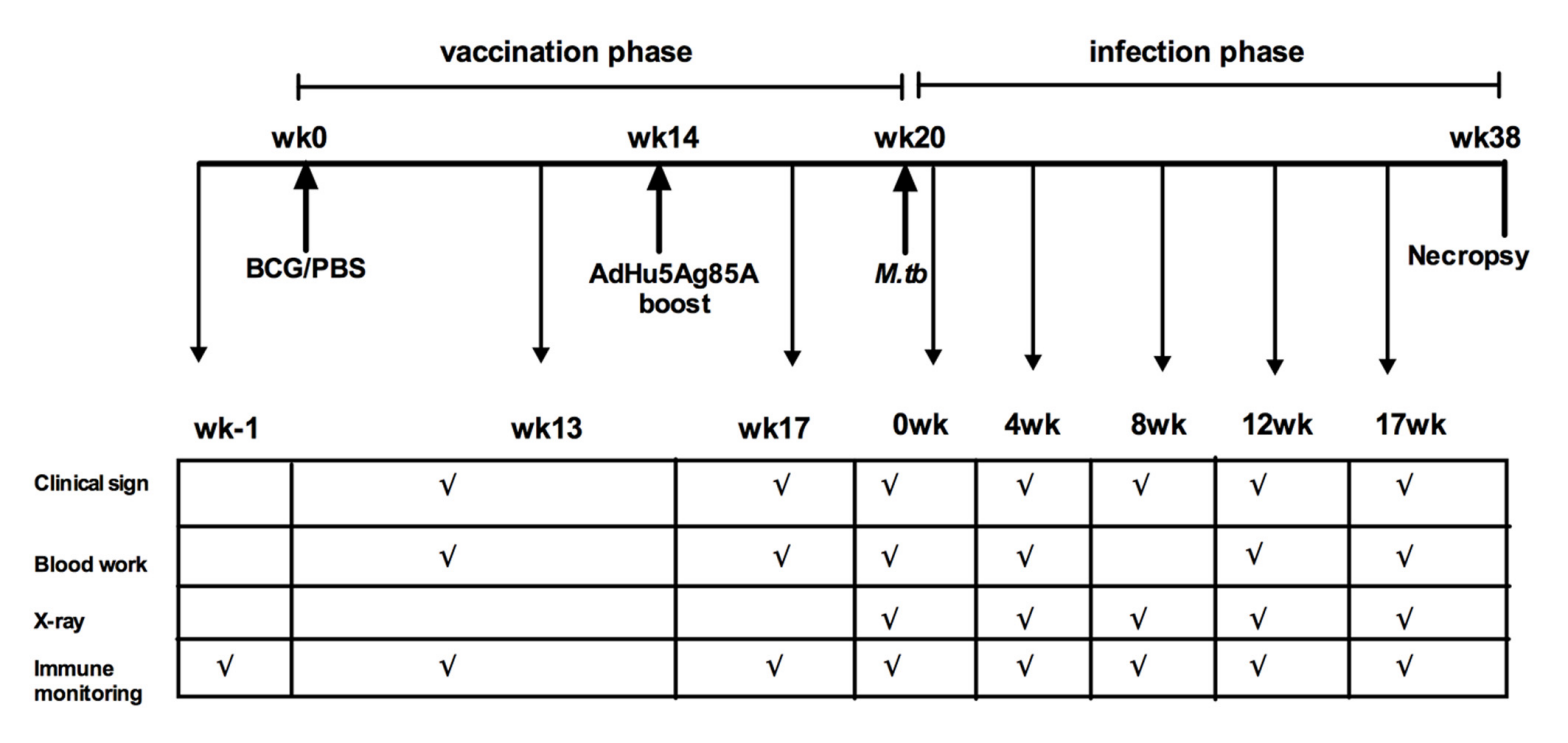
Experimental schema and plan. Depicting the timelines of vaccination (BCG priming at wk0 and AdHu5Ag85A boosting at wk14), *M*.*tb* infectious challenge (wk20) and fixed endpoint/autopsy (wk38). Scheduled times for clinical signs, blood work for acute phase protein measurement, chest X-ray and immune monitoring are indicated. Timelines during infection phase are referred relative to the 18 weeks post-challenge timepoint (indicated within parentheses).

Chest X-ray graphs were acquired at specified time (Fig 1) by using Digi Eye 380 (Mindray). An experienced clinician who was blinded to the treatment groups evaluated the radiographs using a relative scoring system, which yielded scores for each of the upper, middle and lower regions of the right and left lung.

### Clinical assessments

Animals were anesthetized to measure their body weight before and during the infection phase. All the animals were fed 3 times daily; the appetite of each monkey was scored according to the food consumption, i.e. the amount of food taken per meal (2-all; 1-about half; 0-none). A maximum appetite score that an animal could achieve was 6 points per day and 42 per week, which was defined as 100%. A percentage of appetite scores for each animal was calculated as weekly scores divided by 42. Cough score was evaluated on daily basis as well (1-no cough; 0- cough, regardless of cough frequencies). A maximum cough score was 1 point per day and 7 points per week (no cough observed for the week), which was defined as 100%. Blood specimens were collected for the erythrocyte sedimentation rate (ESR), C-reactive protein (CRP), clinical hematology, and biochemistry prior to the inoculation with *M*.*tb* and during the infection phase. Animals that became moribund prior to the study termination endpoint were humanely euthanized. Necropsy was performed within 24 h by experienced veterinary staff.

### Statistical analysis

Data were analyzed by two-tailed non-parametric tests using Graph Pad Prism. Wilcoxon signed rank test was used when comparing measurements at different time points of same animal and Mann-Whitney U test was used when comparing measurements of two different groups. Correlation co-efficient (R) and p-values for R was calculated by non-parametric Spearman’s rho test also using Graph Pad Prism. Results were considered significant for p-values less than or equal to 0.05 and approaching significance for p-value less than 0.1 but greater than 0.05.

## Results

### Respiratory mucosal AdHu5Ag85A boost immunization is safe in parenteral BCG-primed rhesus macaques

Although the safety and efficacy of AdHu5Ag85A delivered via the respiratory mucosal route has been well established and evaluated in a variety of animal models [3,5,21], evaluation of its safety for respiratory mucosal delivery in NHP is a critical step towards human application. To this end, Chinese Rhesus macaques were primed intradermally (i.d.) with BCG and boosted with AdHu5Ag85A at wk14 post-BCG priming via the respiratory mucosal route (i.t. or inhaled aerosol) or parenteral intramuscular route (i.m.) (Table 1 & Fig 1). Control groups of animals were not immunized (naïve–non-v) or received only BCG. Vaccine treatment, dose of vaccine, route of vaccination, and mean weight of animals included in the study are detailed in Table 1. Timelines for immunization and measurements of safety assessments are depicted in the experimental schema (Fig 1). Appetite, acute phase C-reactive protein (CRP), erythrocyte sedimentation rate (ESR) and body temperature (Tem) indicative of systemic inflammation, were measured 3 weeks (wk17) and chest X-ray taken 6 weeks (wk20) post-AdHu5Ag85A boost and before infection (Fig 1). Compared to pre-immunization measurements there were no remarkable changes observed in the appetite, ESR and CRP levels post-immunization (Table 2). No unusual body temperature fluctuations were observed in animals of all treatment groups; and body temperature of animals in BCG/Ad (aerosol) group increased slightly after AdHu5Ag85A boost (Table 2). The chest X-ray remained normal in all boosted groups compared to the controls (Table 2). Taken together, these data suggest that respiratory mucosal delivery of AdHu5Ag85A vaccine is safe in parenteral BCG-primed NHP.

**Table 1:** Characteristics of treatment groups.

	Treatment	Treatment group	Dose	Route	n	Mean weight (kg) (± standard error)
1	non-vaccinated	Naïve	n. a.*	n.a.	4	3.895±0.863
2	BCG vaccinated	BCG	5x10^5^ CFU^$^	i.d.^#^	5	3.998±0.786
3	BCG primed (i.d.) AdHu5Ag85A boosted (it)	BCG/Ad(it)	0.5x10^9^ PFU^@^	i.t.^$$^	6	3.778±0.573
4	BCG primed (i.d.) AdHu5Ag85A boosted (aerosol)	BCG/Ad(aerosol)	2x10^9^ PFU	aerosol	7	4.024±0.547
5	BCG primed (i.d) AdHu5Ag85A boosted (im)	BCG/Ad(im)	1x10^9^ PFU	i.m.^&^	6**	3.987±0.767

n.a.*: does not apply.

i.d.#: intradermal.

i.m.&: intramuscular.

i.t.$$: intratracheal.

CFU$:colony forming unit.

PFU@:plaque forming unit.

**one animal from BCG/Ad(im) group was removed from analysis performed post- infection due to procedure related- accidental death.

**Table 2:** Clinical outcomes post-AdHu5Ag85A boost vaccination.

	BCG/Ad(it)	BCG/Ad(aerosol)	BCG/Ad(im)
Appetite	no change	no change	no change
ESR	no change	no change	no change
CRP	no change	no change	no change
Tem	no change	increased by mean of 0.21C p = 0.05	no change
X-ray*	no change	no change	no change

Note: compared to naïve and BCG alone groups.

*Taken 6 weeks post AdHu5Ag85A boost immunization.

### Respiratory mucosal AdHu5Ag85A boost immunization enhances antigen-specific T cell responses in parenteral BCG-primed rhesus macaques

**Fig 2:**
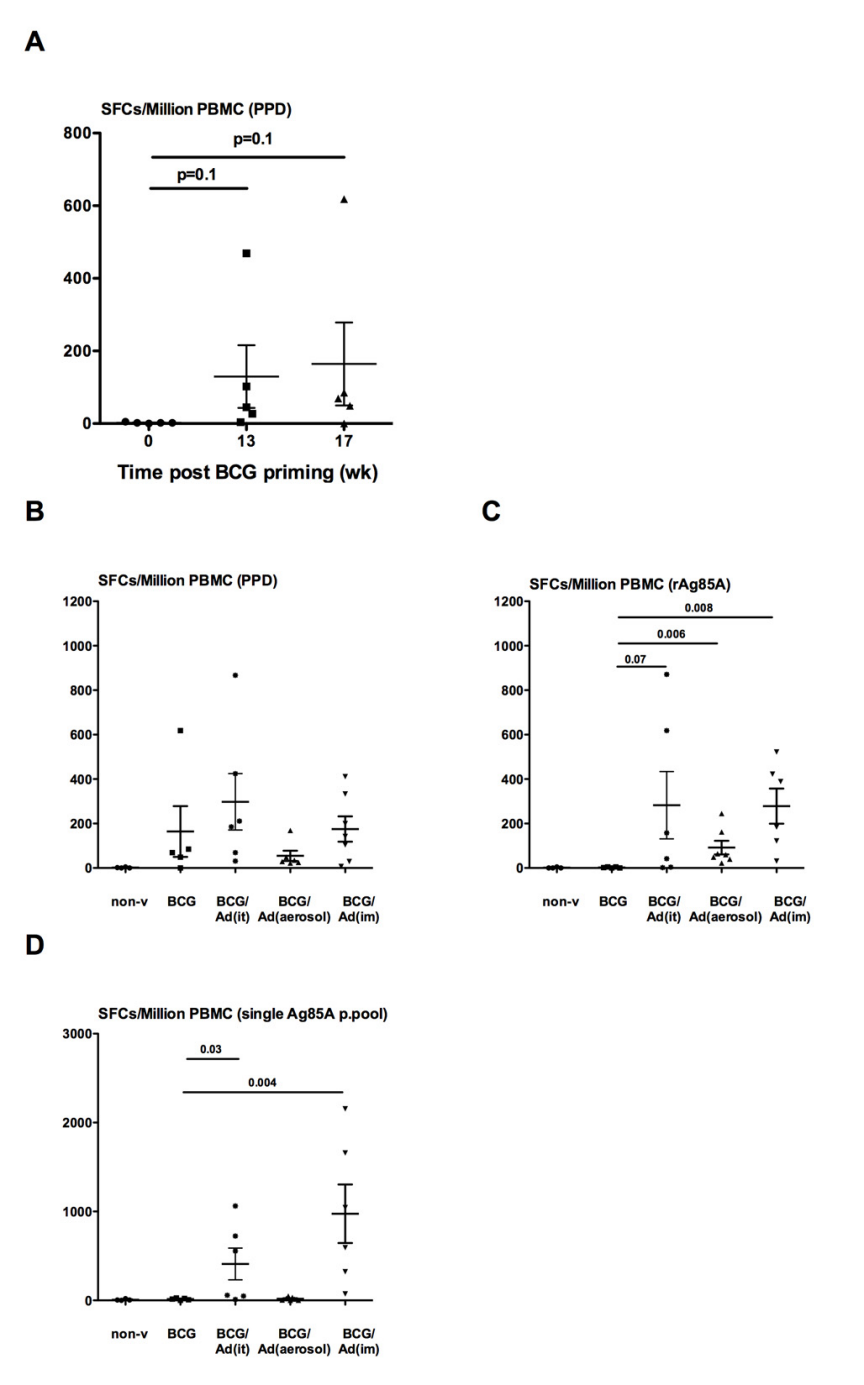
Antigen-specific IFN-γ+ T cell responses post-BCG vaccination or post-BCG prime-Ad boost vaccination. Antigen-specific IFN-γ+ T cell responses in non-boosted BCG-primed animals are measured using IFN- γ ELISOPT assay at 0wk, 13wk and 17wk post BCG vaccination by stimulating fresh peripheral blood mononuclear cells (PBMC) with PPD for 24h (**A**). Scatter dotplot depicting spot forming cells/million PBMC representing the mean responses±standard error. P-values are relative to the time point before BCG vaccination (0wk). (**B/C/D**) depicts antigen-specific IFN-γ responses for each group measured using IFN-γ ELISOPT assay before vaccination (wk0) and after BCG priming (BCG) or after BCG priming and AdHu5Ag85A boosting BCG/Ad(it), BCG/Ad(aerosol), (BCG/Ad(im). Fresh PBMCs are stimulated with PPD (**B**) or rAg85A (**C**) or single pool (**D**) and scatter dotplot depicting mean of spot forming cells/million PBMC ± standard error. P-values above lines indicate comparison between groups. Wilcoxon signed rank test was used to compare measurements at different time points of same animal.

We next investigated the effectiveness of AdHu5Ag85A vaccine to boost parenteral BCG-primed antigen-specific T cell immunity. Rhesus macaques were primed parenterally with BCG and boosted via various routes with AdHu5Ag85A as illustrated in Fig 1. We first verified the immunogenicity of parenteral BCG immunization without boost by ELISPOT assay. Indeed, BCG immunization led to increased responses of the peripheral mononuclear cells (PBMC) to *in vitro* stimulation by *M*.*tb* purified protein derivative (PPD) at both 13 and 17 weeks post-immunization (Fig 2A). Upon examination of the effect of AdHu5Ag85A boost immunization on BCG-primed T cell responses, we found its minimal enhancing effect on PPD-specific responses regardless of the route of boost, compared to non-boosted BCG group (Fig 2B). However, AdHu5Ag85A boost immunization via i.t., aerosol or i.m. route markedly increased T cell responses specific to stimulation by rAg85A protein (Fig 2C) or a single pool of Ag85A peptides (Fig 2D). Likewise, T cell responses to stimulation from the six Ag85A peptide pools, particularly to the pools 1, 2 and 3 (P1, P2 and P3), were significantly increased in AdHu5Ag85A-boosted animals (S1A–S1F Fig). Together these results indicate that respiratory mucosal AdHu5Ag85A boost immunization enhances antigen-specific T cell responses in parenteral BCG-primed animals.

### Respiratory mucosal AdHu5Ag85A boost immunization protects rhesus macaques from pulmonary tuberculosis

**Fig 3:**
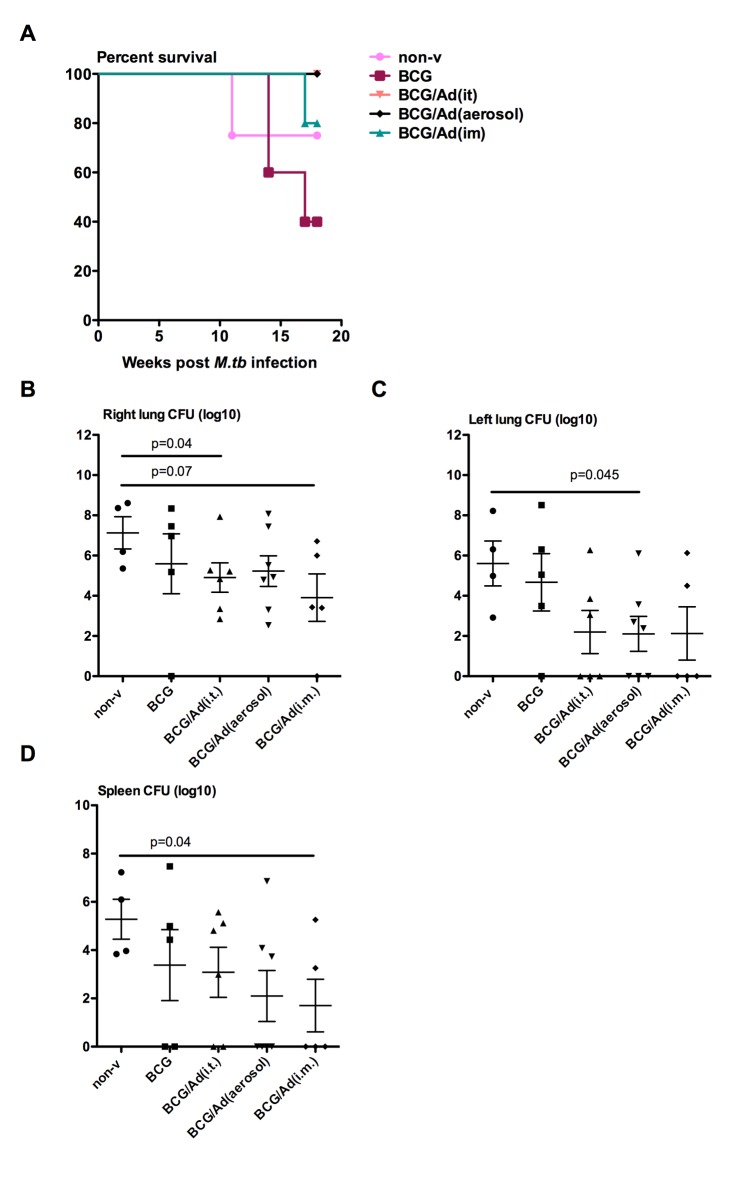
Survival rates and mycobacterial burden in the lung and spleen. Animals were infected with 25 colony-forming units of *M*.*tb* Erdman strain. Group coloring in the survival graph is maintained throughout the article for comparability of data. One animal in BCG/Ad(im) group died accidently and is excluded from data analysis (**A**). Overall log-rank test for trend yielded p = 0.0247, but no statistical differences between groups. Colony-forming units (CFU) of mycobacteria was measured per gram of randomly selected tissue from different lung lobs or spleen at necropsy. CFU per right lung (**B**), left lung (**C**) and spleen (**D**) were presented in logarithmic numbers. P-values above lines indicate comparison between groups. Mann-Whitney rank test is used to compare differences relative to naïve group.

To investigate whether respiratory mucosal AdHu5Ag85A boost immunization could lead to improved immune protection against pulmonary TB, rhesus macaques were parenteral BCG-primed and AdHu5Ag85A-boosted as indicated in Fig 1 and infected via bronchoscopy with a low dose 25 CFU of *M*.*tb* Erdman strain at 6 weeks after boost immunization or 20 weeks after BCG priming (Fig 1). The study termination timepoint was 18 weeks post-infection or 38 weeks post-BCG priming. All animals in the respiratory mucosal-boosted groups (BCG/Ad (it) and BCG/Ad (aerosol)) survived to the study endpoint (Fig 3A). However, one non-vaccinated animal reached the humane endpoint at 11 weeks post-infection whereas three non-boosted BCG-primed animals reached the humane endpoint at 14 (2 animals) and 17 (1 animal) weeks post-infection (Fig 3A). One animal in parenteral-boosted group (BCG/Ad (im)) also reached the humane endpoint at 17 weeks post-infection. All the animals that reached the humane endpoint suffered from health issues related to *M*.*tb* infection confirmed at necropsy and were subject to the measurement of parameters indicated in the experimental plan (Fig 1). One animal in BCG/Ad (im) group was exempt from further examinations due to procedure-related accidental death.

Relative levels of bacterial burden in the lung and spleen were assessed by mycobacterial colony forming assay as a measure of protection. In general, there was reduced bacterial burden in the lungs of all AdHu5Ag85A-boosted groups compared to non-vaccinated naïve or BCG controls (Fig 3B and 3C). In comparison to non-vaccinated naïve control group, while non-boosted BCG immunization only moderately reduced bacterial burden both in the right and left lungs, AdHu5Ag85A boost immunization markedly reduced the overall bacterial burden in the lungs by at least 2 logs. It was particularly apparent in the left lungs of all AdHu5Ag85A-boosted groups where 3 animals in each group remained bacteria-free with the BCG/Ad (aerosol) being significantly different from the naïve control (Fig 3C). In the spleen, the levels of bacteria were also 2–3 logs lower in non-boosted and boosted BCG-primed groups than non-vaccinated naïve controls with the difference seen in BCG/Ad (im) group being significant (Fig 3D). The above data suggest that AdHu5Ag85A boost immunization, particularly the respiratory mucosal boost, enhances protection against pulmonary tuberculosis.

### Respiratory mucosal AdHu5Ag85A boost immunization reduces TB-associated lung pathology in infected rhesus macaques

**Fig 4:**
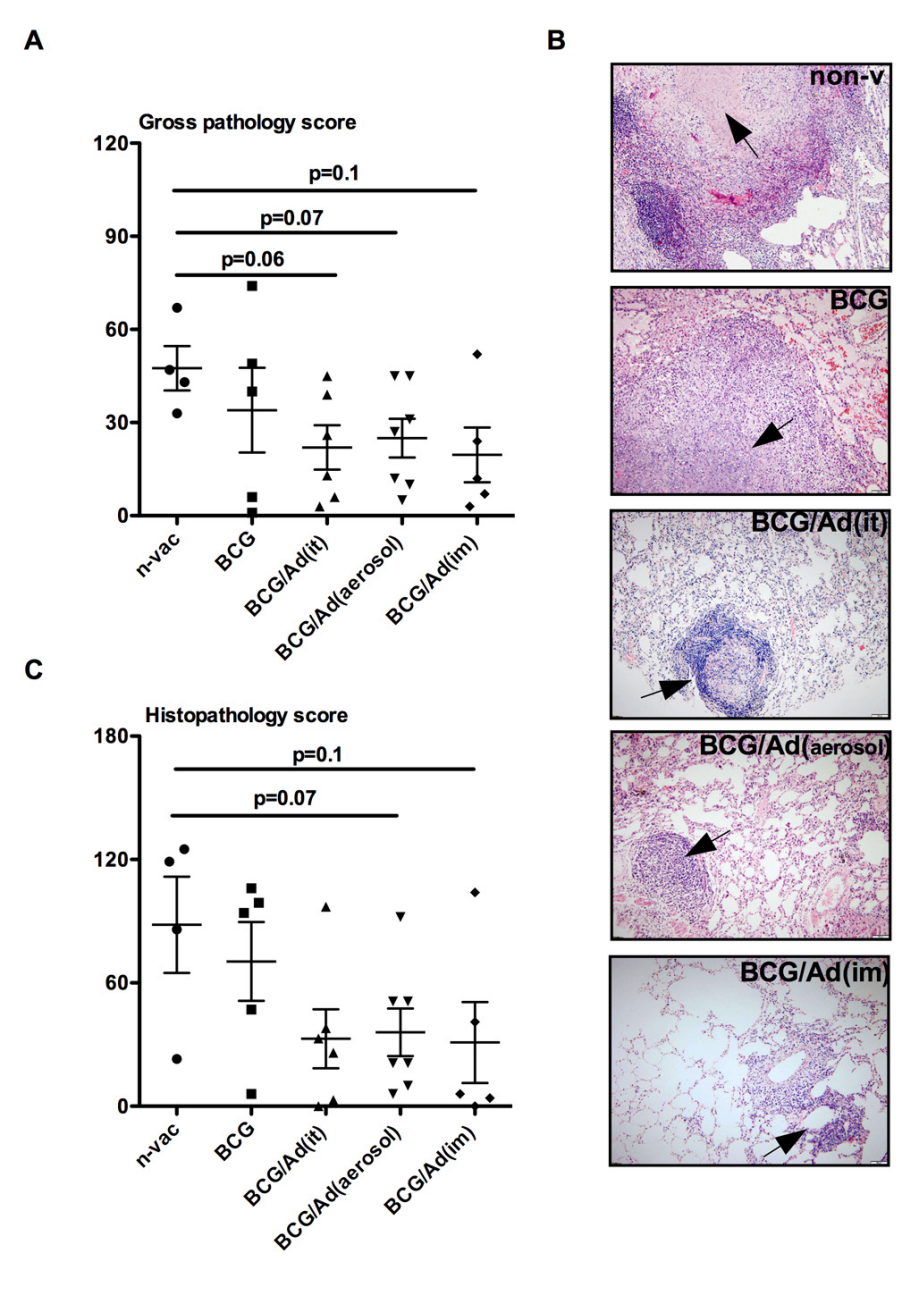
Evaluation of gross pathology and histopathology in M.tb-infected lungs. Individual dots represent scores for animals in each group with mean ± standard error for gross pathology (**A**). (**B**) Representative histomicrographs (x40) of lungs of animals in each group. Arrow points to granuloma with the central caseous necrosis in the lung of non-vaccinated animal (non-v), non-necrotic granuloma in the lung of BCG vaccinated animal (BCG), scattered small size granulomas lacking necrosis in the of lung of AdHu5Ag85A boosted animals (BCG/Ad(it), BCG(aerosol) and BCG(im)). (**C**) Individual dots represent scores for animals in each group with mean± standard error for histopathology. Mann-Whitney rank test is used to compare differences relative to naïve group.

We next assessed the gross pathological and histopathological changes in the lung. The lungs of non-vaccinated controls and non-boosted BCG-primed animals displayed severe gross pathology including consolidation, irregular discolorization and scattered nodular lesions (S2 Fig). Such severe gross pathology was observed in four out of 4 non-vaccinated controls and two out of 5 non-boosted BCG-primed animals. By comparison, the lungs of AdHu5Ag85A-boosted animals had overall reduced gross pathology (S2 Fig). One out of 5 AdHu5Ag85 i.m. boosted animals displayed severe gross pathology, failing to reach the study termination timepoint while one displayed a large lesion in the right lung with the remaining animals free of large gross pathologic lesions in their lungs. Only one out of 6 AdHu5Ag85A i.t. boosted animals displayed large lesions in the right lung with other 5 animals remaining free of visible gross lesions. Four out of 6 AdHu5Ag85A aerosol boosted animals displayed scattered lesions in their lungs. Thus, only the lungs of respiratory mucosal AdHu5Ag85A-boosted animals (BCG/Ad(it) and BCG/Ad(aerosol)) had markedly reduced overall gross pathological scores approaching significance (p<0.1) compared to non-vaccinated naïve controls (Fig 4A).

In keeping with reduced gross pathology, histopathological examination revealed striking differences in microscopic granulomatous lesion size, necrosis and inflammation between non-vaccinated naïve/non-boosted BCG-primed animals and AdHu5Ag85A-boosted animals (Fig 4B). The lungs of the non-vaccinated naïve and non-boosted BCG-primed animals had a multitude of large granuloma lesions but the centre of the lesions in naïve animals was often necrotic whereas it was not in non-boosted BCG group. In contrast, respiratory mucosal-boosted as well as i.m-boosted animals had markedly reduced overall inflammation and the granulomas were scattered and much smaller in size with intense lymphocytic infiltration (Fig 4B). Thus, the histopathologic scores in these animals were significantly reduced compared to naïve or non-boosted BCG-primed counterparts (Fig 4C). Taken together, the above data establish that respiratory mucosal AdHu5Ag85A boost immunization markedly reduces TB-associated histopathologic lesions in the lung of parenteral BCG-primed rhesus macaques.

### Improved clinical outcomes by respiratory mucosal AdHu5Ag85A boost immunization in infected rhesus macaques

**Fig 5:**
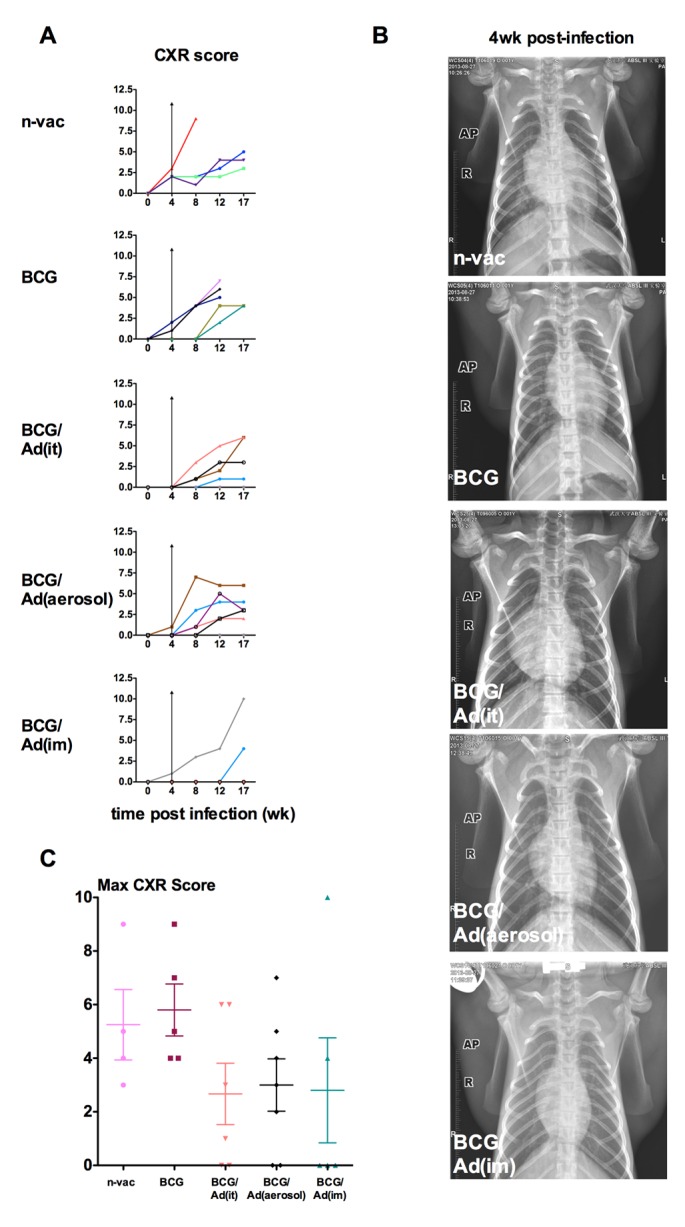
Longitudinal kinetics of radiological changes in the lung. Chest X-ray (CXR) was acquired by using DIGI Eye 380 by placing anesthetized animals in dorsal recumbence position. X-ray images were scored using relative scoring system. (**A**) Lines represent individual animals in each group and color assigned to individual animals in each group is kept consistent throughout the article for comparability. Wilcoxon ranked test is used to compare CXR scores post-infection relative to values before infection (0), showing mean. Vertical arrow lines in the graphs indicate the evidence of CXR changes 4 weeks post-infection in non-vaccinated naïve and non-boosted BCG-primed animals, but not in AdHu5Ag85A-boosted animals. (**B**) Representative radiographs of CXR of animals in each group 4 week post-infection. (**C**) Scatter dotplot depicting the mean maximum CXR scores±standard error for animals in each group. Mann-Whitney rank test is used to compare CXR scores in vaccinated groups to naïve group.

Radiological examination is a standard clinical diagnostic means to assess lung pathology in active TB, allowing the kinetic observation in real time. We next examined the chest X-ray (CXR) radiographs taken during the infection phase (Fig 1) and quantified lung pathologic changes by using a defined scoring mechanism. In comparison to non-vaccinated naïve and non-boosted BCG-primed animals, AdHu5Ag85A-boosted animals displayed delayed CXR changes post-infection (Fig 5A–5C). While the CXR changes were evident at 4 weeks post-infection in non-vaccinated naïve and non-boosted BCG-primed animals, the majority of AdHu5Ag85A-boosted animals remained disease-free until 8 weeks post challenge (Fig 5A and 5B). Furthermore, both respiratory mucosal and parenteral boost immunization also markedly reduced the overall maximal CXR scores with only 25% of AdHu5Ag85A-boosted animals scoring above 4 compared to 75% of non-vaccinated naïve and non-boosted BCG-primed animals displaying severe lung pathology (score >4) (Fig 5C).

**Fig 6:**
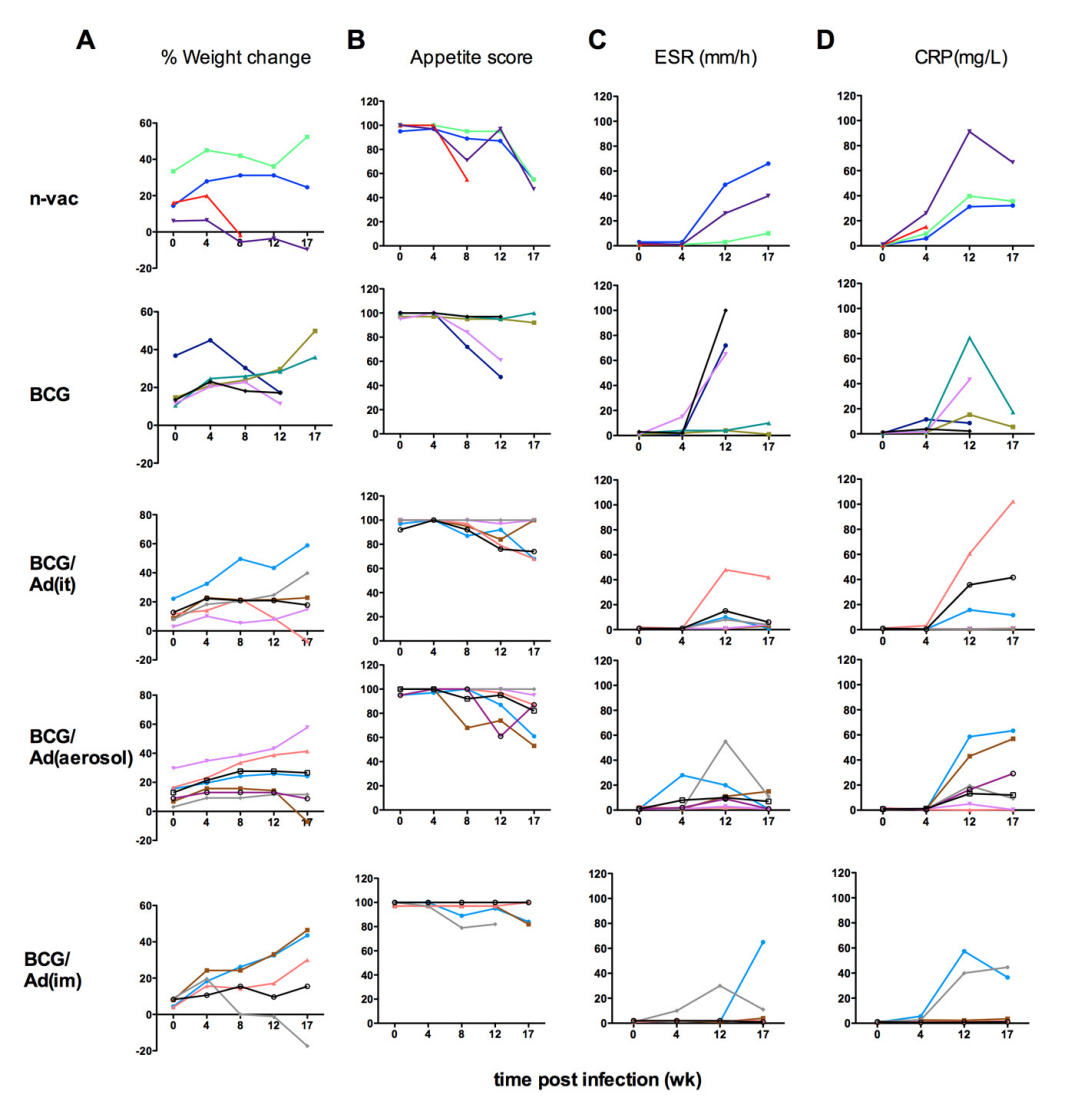
Longitudinal kinetics of clinical outcomes during the course of infection. Clinical outcomes were monitored at scheduled times as indicated in the experiment plan and as described in Methods. Line graphs depict (**A**) percent of weight change relative to weight at the start of the experiment, (**B**) appetite, (**C**) change in erythrocyte sedimentation rate (ESR), and (**D**) C-reactive protein (CRP). Lines represent individual animals in each group and color assigned to individual animal in each group is maintained to display each clinical sign evaluated for comparability. Wilcoxon ranked test is used to compare clinical signs post infection relative to values before infection.

There was an overall improvement in other clinical manifestations including body weight changes, appetite, ESR and CRP by AdHu5Ag85A boost immunization (Fig 6A–6D). For instance, >50% of non-vaccinated naïve controls and non-boosted BCG-primed animals showed progressive weight losses and more prominent appetite loss over the infection phase whereas most AdHu5Ag85A-boosted animals demonstrated a sustained or gaining trend in body weight and less severe appetite losses (Fig 6A and 6B). Upon measuring the ESR and CRP values in the peripheral blood as indices of the severity of TB, we found that the overall rises in both ESR and CRP were delayed in AdHu5Ag85A-boosted animals compared to the naïve or non-boosted BCG-primed animals (Fig 6C and 6D). Furthermore, analysis of cough scores revealed significant reduction in the frequency of cough in AdHu5Ag85A aerosol-boosted animals from 12 weeks post-infection (data not shown). The above data collectively suggest that consistent with the protection data, respiratory mucosal AdHu5Ag85A boost immunization improves TB disease-related clinical manifestations of infected rhesus macaques.

### Lung histopathology directly correlates with bacterial burden and clinical outcomes but inversely correlates with post-infection Ag85A-specific T cell responses

**Fig 7:**
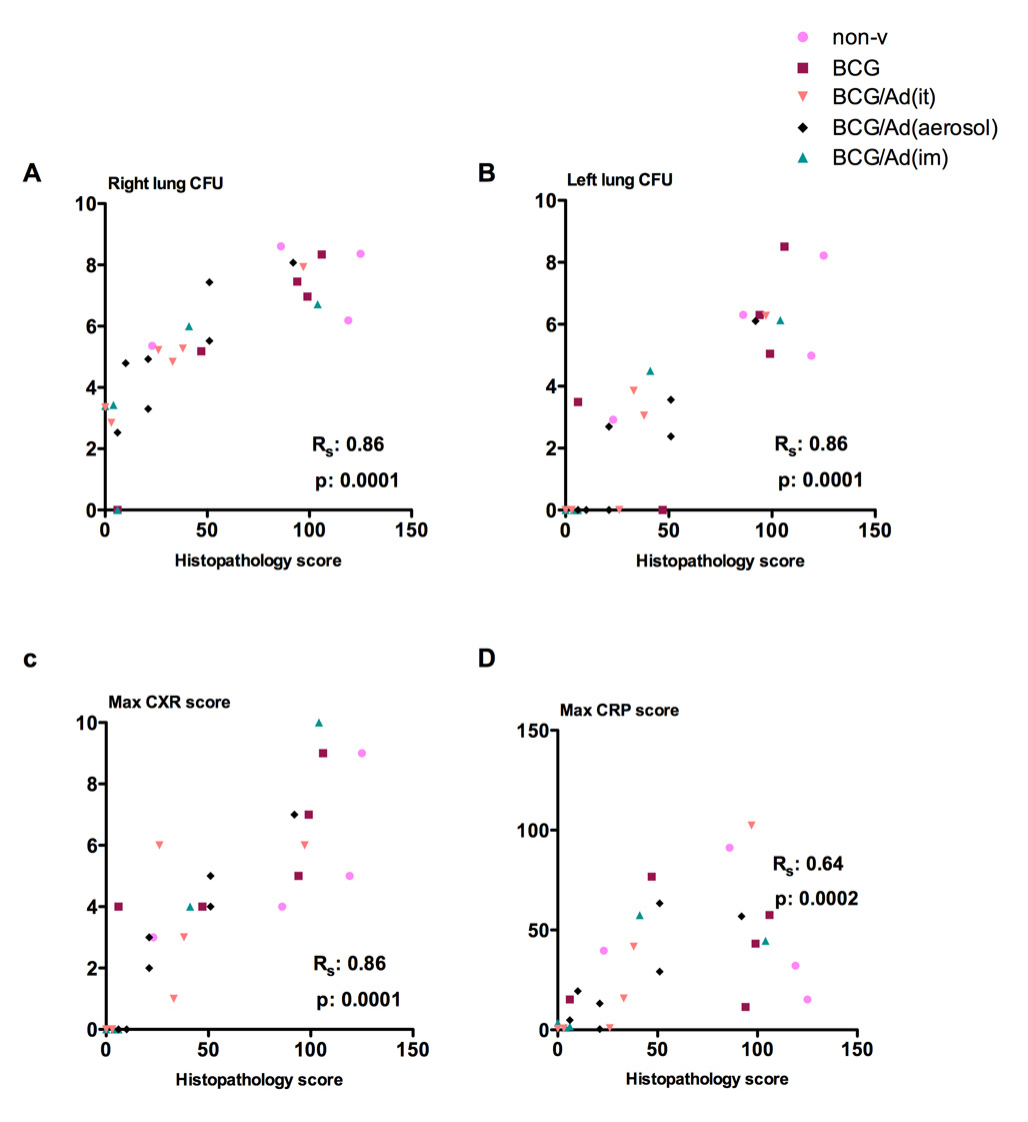
Correlation of lung histopathology with microbiological and clinical outcomes. Bacterial burden in the lung, chest radiography (CXR) and C-reactive protein (CPR) levels in blood correlate significantly with lung histopathology. Parameters are plotted (maximum values for each of the parameters) per individual animal (color coordination consistent throughout the article) against lung histology score for right lung (**A**), left lung bacterial burden (**B**), CXR (**C**), and CRP (**D**). Spearman’s rho (Rs) correlation factors and p-values are indicated.

To assess the protective correlates in NHP models of TB vaccination, we first analyzed the correlation between lung histopathology and bacterial burden as well as clinical outcomes (Fig 7). Co-efficient of correlation between individual maximum histopathological scores and maximum bacterial counts in the right lung (Fig 7A) and left lung (Fig 7B) was determined. There was a highly significant correlation between lung histopathology and lung bacterial burden (Fig 7A and 7B). Similarly, lung histopathology was also directly significantly correlated with individual maximum CXR scores and maximum CRP values (Fig 7C and 7D).

**Fig 8:**
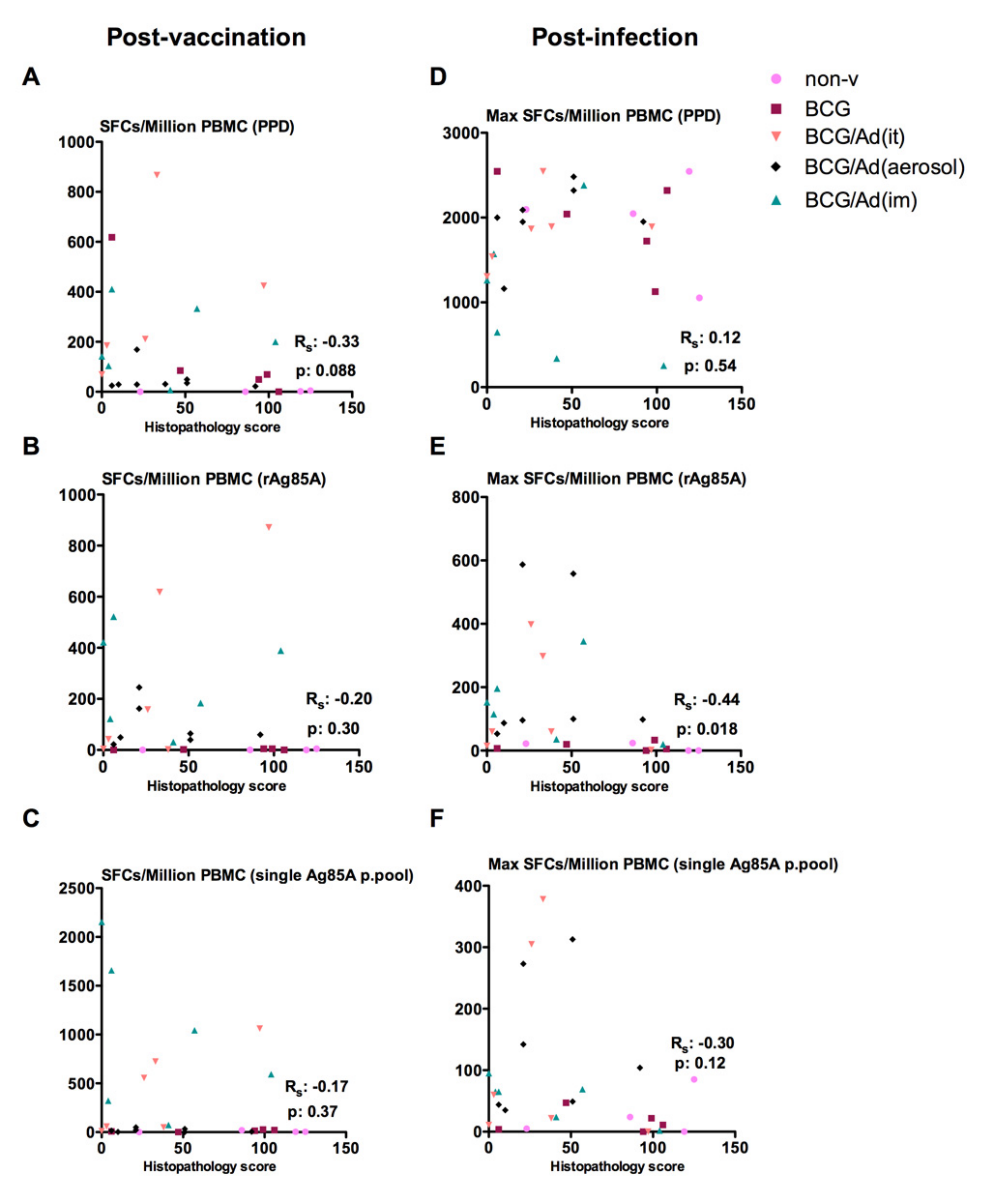
Correlation of vaccine-activated T cell responses with lung histopathology post-vaccination and post-infection. Maximal PPD-specific PBMC IFN-γ responses post-vaccination and maximal rAg85A-specific IFN-γ responses post-infection show significant inverse correlations with histopathology in the lung. Maximal antigen-specific PBMC responses for PPD (**A**), rAg85A (**B**) and single pool (**C**) post-vaccination are plotted per individual animal against histopathological scores. Maximal antigen-specific PBMC responses for PPD (**D**), rAg85A (**E**) and single pool (**F**) post-infection are plotted per individual animal against histopathological scores. Spearman’s rho (Rs) correlation factors and p-values are indicated.

Immune protective correlates still remain poorly understood in vaccinated humans and NHP. Since we observed a strong correlation between lung histopathology and TB-associated clinical outcomes, we next evaluated the relationship between lung histopathology and Ag-specific T cell responses before (post-vaccination) and after infection (post-infection). Individual maximum PPD-specific immune responses, but not rAg85A- or single Ag85A peptide (p.) pool-specific responses, inversely correlated with lung histopathology before infection (Fig 8A–8C). In contrast, rAg85A- or single Ag85A p.pool-specific immune responses, but not PPD-specific responses, inversely correlated with lung histopathology after infection (Fig 8D–8F). Post-infection kinetic of PPD-specific responses progressively increased in non-vaccinated and vaccinated animals (S3A Fig). Contrarily, rAg85A and single Ag85A p.pool-specific responses progressively decreased (S3B and S3C Fig) in AdHu5Ag85A boosted animals. These data together suggest that AdHu5Ag85A vaccine-activated Ag85A-specific responses may play a role in immune protection against pulmonary tuberculosis in infected rhesus macaques.

## Discussion

Mounting evidence suggests it would be advantageous for a novel TB vaccine to elicit anti-TB immunity at the respiratory mucosa to exert immediate local protection upon pathogen entry [3,5,6,7,8,9]. AdHu5Ag85A, a novel tuberculosis vaccine developed as a boost vaccine to enhance BCG-mediated immunity has been shown to be safe for respiratory mucosal delivery and to elicit potent anti-TB immunity in a variety of animal models [3,5,21]. However, its safety and protective efficacy following respiratory mucosal delivery has not been previously evaluated in non-human primates. Given NHP’s high degree of genomic and disease similarity to humans, vaccine testing in NHP represents a critical step towards its further evaluation and application in humans [13,15,16,18,19]. While recently the MVA- and AdHu35-based candidate TB vaccines have been evaluated in NHP following respiratory mucosal delivery, none of these candidates was shown to enhance protection [19,20]. Here we demonstrate that respiratory mucosal boosting by intratracheal (it) inoculation or inhaled aerosol of AdHu5Ag85A was safe and well-tolerated in parenteral BCG-primed rhesus macaques. We also show that AdHu5Ag85A respiratory mucosal boosting enhanced Ag85A–specific T cell responses. Of importance, for the first time we provide the evidence that respiratory mucosal boosting significantly improved the protective efficacy of parenteral BCG priming in NHP, including improved survival rates and bacterial control following pulmonary *M*.*tb* Erdman infection. Furthermore, respiratory mucosal boosting reduced TB-associated lung histopathology and improved a number of other relevant clinical outcomes in infected BCG-primed rhesus macaques.

In addition to its potency in inducing quality mucosal immunity, another characteristic of respiratory mucosal delivery technology such as aerosol delivery is needle-free, a feature highly desired for human immunization [26]. However, compared to the parenteral route of delivery, safety is a more important consideration for respiratory mucosal delivery. For instance, respiratory mucosal inoculation of AdHu5-based vaccine may cause undesired tissue inflammation. With the success in human intranasal Flumist immunization program and aerosol measles vaccine studies, human respiratory mucosal TB vaccination has become a promising modality [27]. This conviction is further supported by a recent phase 1 human study showing the safety and immunogenicity of MVAAg85A TB vaccine delivered via aerosol [13]. Human adenovirus serotype 5, the backbone of AdHu5Ag85A, has an excellent safety record for human use [28]. Indeed in the current study we found no major adverse effects associated with either direct intratracheal or aerosol delivery of AdHu5Ag85A. Thus, its safety and enhanced protection against pulmonary TB by a single respiratory mucosal delivery in rhesus macaques supports its great potential for human application and further clinical investigation.

Although in general viral-vectored vaccines are amenable for respiratory mucosal immunization because of their natural tropism, vaccine efficacy by different viral vectors may vary and depend on their respective inherent properties. For instance, it has recently been shown that despite robust T cell responses elicited by aerosol AERAS-402 (an Ad35-based TB vaccine), it failed to provide enhanced protection [19]. This is likely in part due to the induction of type I IFN by Ad35-based vector system [29]. Indeed, Type I IFNs have been shown to inhibit macrophage activation, thus discouraging mycobactericidal activities during Th1 immunity [30,31,32]. Furthermore, different viral vectors may also vary in their immunogenicity. In this regard, among many human Ad vectors Ad5 is the most highly immunogenic. Thus a single dose of AdHu5Ag85A potently boosted BCG-induced immunity [12] whereas three doses of AERAS-402 were required to boost BCG-induced immunity [11].

There is an urgent need to identify immune protective correlates following TB vaccination in humans [2]. Use of NHP models also provides the opportunity for identification of potential biological signatures that may help predict the protective efficacy in vaccine trials. The long-held belief is that T cell IFN-γ responses serves as biomarker of protection. In our current study the immune-monitoring post-vaccination in the peripheral blood reveals that IFN-γ+ T cell responses in the peripheral blood were less prominent in the animals boosted via the respiratory mucosal route than via the parenteral intramuscular route. This could be attributed to specific mucosal homing and localization of antigen-specific T cells to the lung, the site of immunization. This is in agreement with previous findings showing a correlation between geographical localization of Ag-specific T cell responses and the route of delivery in a macaque TB vaccine study [20]. In agreement with the current view that the magnitude of vaccine-induced specific T cell responses prior to *M*.*tb* challenge correlates positively with immune protection, we found an inverse correlation between maximal PPD-specific T cell responses and lung histopathological scores. However, post-infection levels of PPD-specific T cell responses no longer correlated with histopathological changes. Rather, post-infection maximal rAg85A- and single Ag85A p-pool-specific responses were inversely correlated with lung histopathological scores. Of note, rAg85A- and single Ag85A p-pool-specific responses were higher in AdHu5Ag85A-boosted animals compared to non-vaccinate and BCG alone-vaccinated animals post-infection. With the AdHu5Ag85A specifically designed to boost the memory T cells specific for Ag85A in BCG vaccinees, our findings suggest that immune monitoring of Ag85A-specific T cell responses in the circulation may represent a biomarker of vaccine efficacy in clinical trials.

Two recent studies on aerosol TB vaccine studies in NHP models used rhesus macaques of Indian origin [19,20]. In the current study we have used rhesus macaques of Chinese origin. It is known that susceptibility to TB may differ between different origins of NHP and rhesus macaques of Indian and Chinese origins display marked genetic differences [33]. Thus, the origin of macaques, challenge dose and virulent nature of *M*.*tb* are relevant to modeling tuberculosis for vaccine evaluation studies. A low-dose challenge of 25 CFU of *M*.*tb* Erdman strain was chosen in our current study based on the observation from a recent study showing that this dose can recapitulate acute human TB disease [22]. Our findings of TB-associated pathology in non-vaccinated control macaques are in agreement with the findings described by Zhang et al [22]. It is noteworthy that in our current study BCG vaccination alone only modestly reduced bacterial burden. This supports the findings from a previous study where BCG immunization had a minimal protective effect in rhesus macaques challenged with *M*.*tb* Erdman [34]. Despite insignificantly reduced CFU, we did find that BCG immunization reduced granuloma necrosis compared to severe necrotized granuloma seen in unvaccinated control macaques.

In summary, we have demonstrate the safety and improved protection following a single dose of AdHu5Ag85A boosting vaccine delivered via the respiratory mucosal route in parenteral BCG-primed NHP. Our findings warrants further clinical evaluation of this candidate vaccine for respiratory mucosal delivery in BCG-vaccinated humans. Our study also provides the evidence to support the development of mucosal vaccination strategies against other respiratory intracellular pathogens

## Supporting Information

S1 Fig Ag85A peptide pool specific-PBMC responses post-AdHu5Ag85A boost vaccination. Antigen-specific IFN-γ responses to six pools (P1-P6) each pool containing 10 of Ag85A peptide (each peptide with 7 to 10 overlapping amino acids) of individual animal measure by ELISPOT at 3 week post-AdHu5Ag85A boost (BCG/Ad(it), BCG/Ad(aerosol), BCG/Ad(im) or 17wk post-BCG priming (BCG). Scatter dotplot depicting the mean of spot forming cells/million PBMC ± standard error.
(TIFF)

S2 Fig Gross pathology of *M*.*tb-*infected lung. Representative lung images from each group of animals showing gross pathological changes in the lung at necropsy. The lungs of non-vaccinated controls (non-v) and non-boosted BCG-primed animals (BCG) display severe gross pathology including consolidation, irregular discolorization and scattered nodular lesions. In comparison, gross pathological changes in the lung are markedly reduced in AdHu5Ag85A-boosted animals (BCG/Ad(it), BCG/Ad(aerosol), BCG/Ad(im)).
(TIF)

S3 Fig Longitudinal kinetics of antigen-specific PBMC responses during the course of infection. Antigen-specific IFN-γ responses are measured using IFN-γ ELISOPT assay during the infection phase. Fresh peripheral blood mononuclear cells are stimulated with PPD (**A**) or rAg85A (**B**) or single peptide pool (**C**). Line graphs depict SFCs/million at specified times post-infection expressed as the mean of SFCs ± standard error.
(TIF)
