# Supplementary figures and images for: AdHu5Ag85A Respiratory Mucosal Boost Immunization Enhances Protection against Pulmonary Tuberculosis in BCG-Primed Non-Human Primates
Source: PLoS One. 2015 Aug 7;10(8):e0135009. doi: 10.1371/journal.pone.0135009 (PMC4529167; doi:10.1371/journal.pone.0135009)

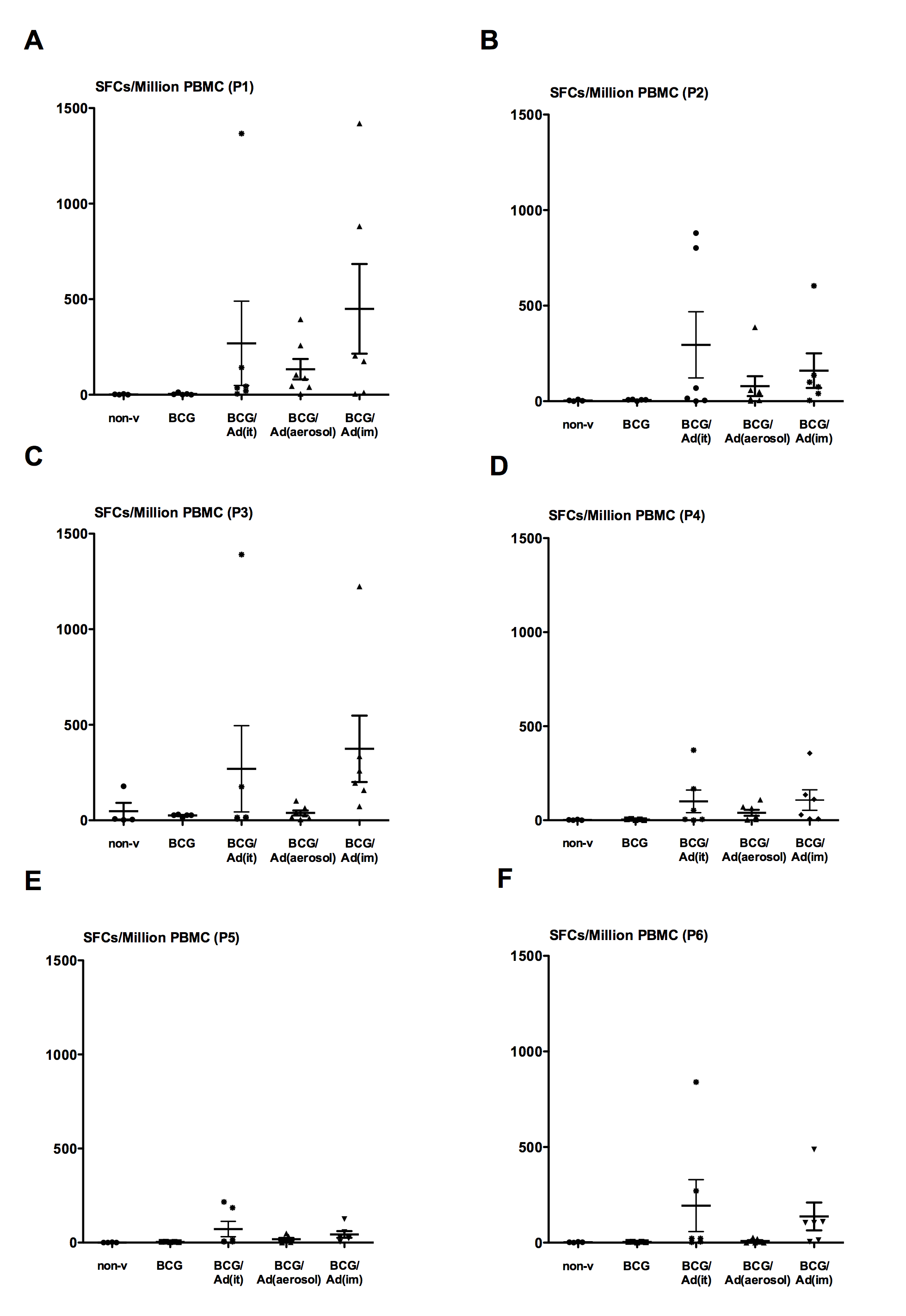

Supplement: S1 Fig — Antigen-specific IFN-γ responses to six pools (P1-P6) each pool containing 10 of Ag85A peptide (each peptide with 7 to 10 overlapping amino acids) of individual animal measure by ELISPOT at 3 week post-AdHu5Ag85A boost (BCG/Ad(it), BCG/Ad(aerosol), BCG/Ad(im) or 17wk post-BCG priming (BCG). Scatter dotplot depicting the mean of spot forming cells/million PBMC ± standard error. (TIFF) [file pone.0135009.s001.tiff]

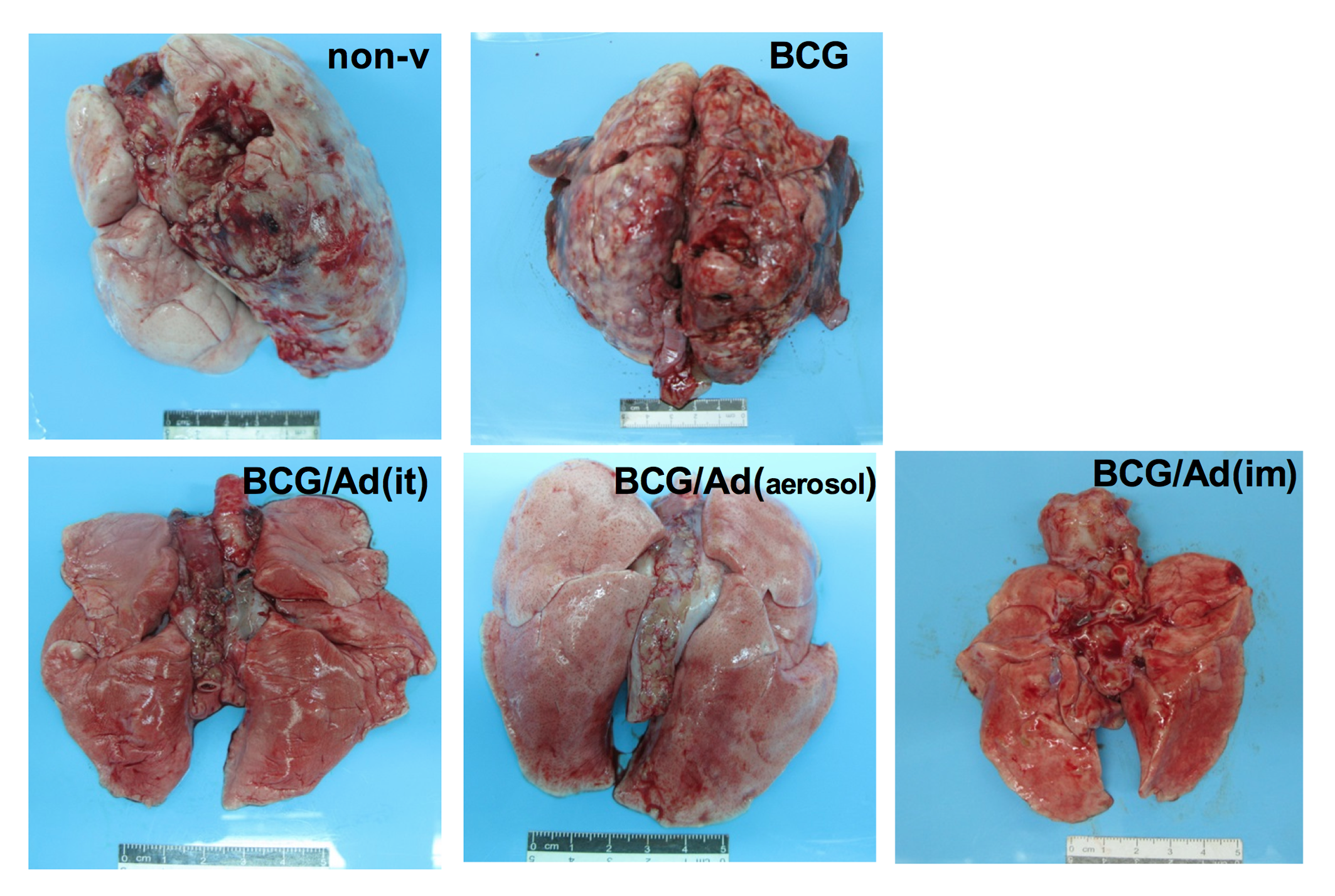

Supplement: S2 Fig — Representative lung images from each group of animals showing gross pathological changes in the lung at necropsy. The lungs of non-vaccinated controls (non-v) and non-boosted BCG-primed animals (BCG) display severe gross pathology including consolidation, irregular discolorization and scattered nodular lesions. In comparison, gross pathological changes in the lung are markedly reduced in AdHu5Ag85A-boosted animals (BCG/Ad(it), BCG/Ad(aerosol), BCG/Ad(im)). (TIF) [file pone.0135009.s002.tif]

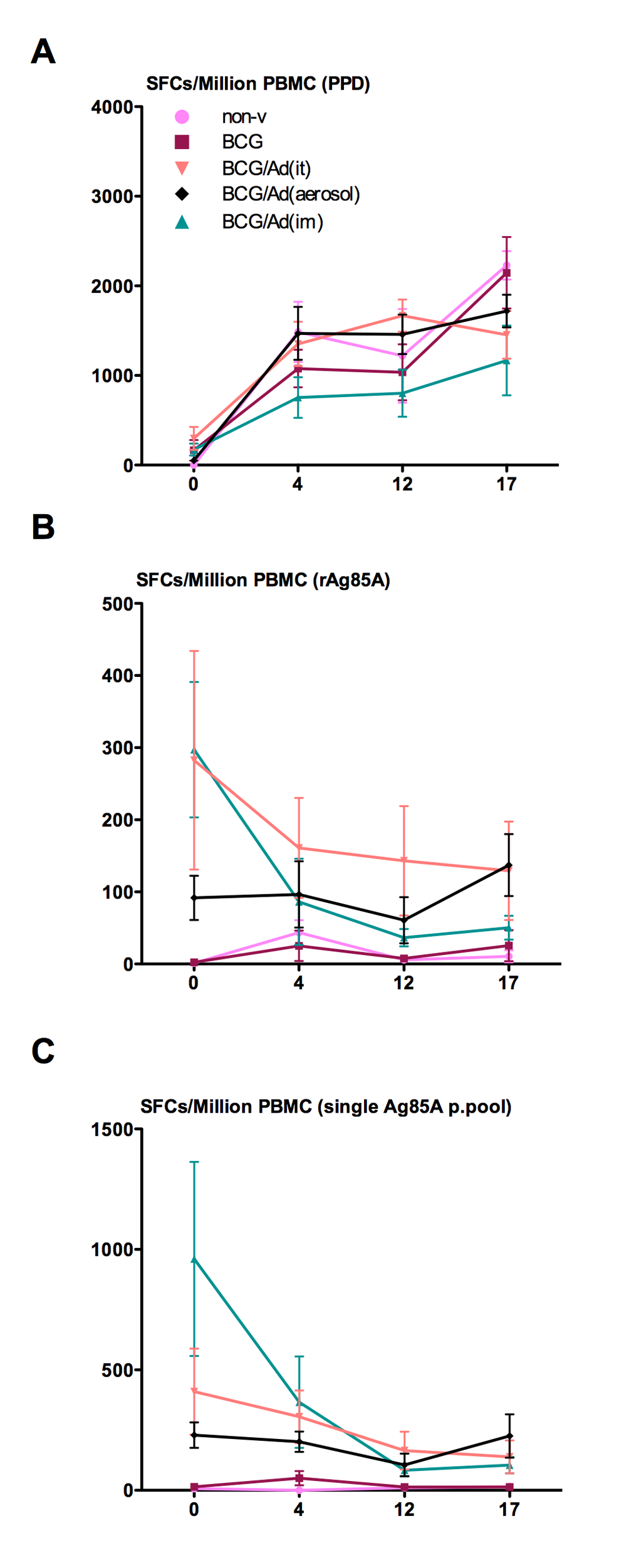

Supplement: S3 Fig — Antigen-specific IFN-γ responses are measured using IFN-γ ELISOPT assay during the infection phase. Fresh peripheral blood mononuclear cells are stimulated with PPD (A) or rAg85A (B) or single peptide pool (C). Line graphs depict SFCs/million at specified times post-infection expressed as the mean of SFCs ± standard error. (TIF) [file pone.0135009.s003.tif]
